# Neurally adjusted ventilatory assist in critical care patients with and without obesity: a prospective randomized crossover study

**DOI:** 10.1186/s13613-025-01552-x

**Published:** 2025-08-29

**Authors:** Matthieu Conseil, Samir Jaber, Fabrice Galia, Nicolas Molinari, Gerald Chanques, Audrey De Jong, Mathieu Capdevila

**Affiliations:** 1 Department of Anaesthesiology and Critical Care Medicine B (DAR B), Saint-Eloi Hospital, University Teaching Hospital of Montpellier, 80 avenue Augustin Fliche, Montpellier, 34295 France; 2 INSERM U1046 PhyMedExp, Montpellier, France; 3 Department of Statistics, University of Montpellier Lapeyronie Hospital, UMR 729 MISTEA, 371 Av. du Doyen Gaston Giraud, Montpellier, 34090 France

**Keywords:** Nava, Obesity, Weaning, Mechanical ventilation

## Abstract

**Background:**

Neurally Adjusted Ventilatory Assist (NAVA) compared to Pressure Support Ventilation (PSV) improves patient-ventilator interactions in intensive care unit. No study has evaluated NAVA in patients with obesity. We aimed to assess the feasibility and safety of NAVA in patients with obesity, and to compare NAVA in patients with versus without obesity.

**Methods:**

In this randomized cross-over study, all respiratory cycles during 1 h of mechanical ventilation from 10 patients with obesity and 11 without obesity were analyzed. Patients underwent 30 min of NAVA and 30 min of PSV in a random order. Flow, airway pressure and diaphragm electrical activity were continuously recorded. Arterial blood gases were obtained at baseline and at the end of each 30-min period. Patient-ventilator interactions were assessed with trigger delay, inspiratory time in excess, rate and type of dyssynchrony cycles. Variability of the ventilatory parameters was evaluated by the coefficient of variation (SD/mean).

**Results:**

All patients concluded the study, with a total of 1790 ± 873 respiratory cycles analyzed per patient. In patients with obesity, NAVA versus PSV was associated with a significant reduction in trigger delay (0 [0–5] vs. 106 [34–125] ms, *p* < 0.05), inspiratory time in excess (96 [94–102] vs. 145 [137–202] ms, *p* < 0.01) and in ineffective efforts (0 [0-0.03] vs. 0.33 [0.23–0.37] events/min, *p* < 0.05). The global dyssynchrony index remained similar in both modes (2.2% [1.1–4.4] vs. 3.7% [2.4–5.6], *p* = 0.68). Compared to PSV, PaO2/FiO2 ratio significantly increased in NAVA, 238 mmHg [174–344] versus 207 mmHg [164–297], *p* < 0.05. The tidal volume was significantly lower during NAVA than during PSV, 6.7 mL/kg predicted body weight [5.9–7.1] versus 7.2 mL/kg [6.2–8.2], *p* < 0.05. Ventilatory variability was significantly higher with NAVA, 16% [11-21] versus 4% [2-4] in mean inspiratory airway pressure. These results were similar in patients without obesity and the obesity factor was never significant. No adverse event was observed in patients with and without obesity in both modes.

**Conclusion:**

In patients with obesity, NAVA ventilation is feasible and safe, improves patient-ventilator interactions and oxygenation, with an increase ventilatory variability compared to PSV. The effects of NAVA are comparable in patients with and without obesity.

**Supplementary Information:**

The online version contains supplementary material available at 10.1186/s13613-025-01552-x.

## Introduction

Pressure Support Ventilation (PSV) is the most used mode during the weaning of mechanical ventilation in intensive care unit [1, 2]. However the patient’s ventilatory demand is variable and this mode provides a fixed level of pressure, which may lead to a mismatch between the patient’s request and the level of assistance. Neurally Adjusted Ventilatory Assist (NAVA) is a mode of mechanical ventilation where the diaphragm electrical activity (EADi) controls the timing and the amount of assistance [3]. In critically ill patients NAVA can improve patient-ventilator synchrony [4, 5, 6], respiratory variability [7, 8], oxygenation [8], and it can decrease the duration of mechanical ventilation [9]. In situation of difficult weaning, NAVA could be a more appropriate mode than PSV [10]. These benefits were also confirmed with noninvasive ventilation, in chronic obstructive pulmonary disease exacerbation [11], during acute respiratory failure [12] and after extubation [13].

Over the past years, there has been a significant increase in obesity rates worldwide [14] and the prevalence of patient with obesity is increasing in intensive care units [15, 16]. Patients with obesity have impaired lung function with decreased functional residual capacity and compliance, increased airway resistance and ventilation-perfusion mismatch [17]. These changes are causing an increased incidence of complications and a longer duration of weaning from mechanical ventilation in intensive care [17]. Specific respiratory management is required during weaning and after extubation for these patients [18, 19, 20].

However, diaphragmatic electromyogram (EMG) acquisition, necessary for NAVA, could be compromised by increased fatty infiltration of the respiratory muscles and pleura [21, 22]. Signal acquisition could also be affected by the distance between diaphragm and esophagus [23] related to obesity. To our knowledge no physiological study has been performed to evaluate the use of NAVA in patients with obesity.

The hypothesis of the present study is that NAVA use is feasible and safe in patients with obesity.

The first objective of this study was to evaluate the safety and the effectiveness (ventilator parameters, gas exchange and dyssynchronies) of NAVA in patients with obesity. The secondary objective was to compare NAVA in patients with and without obesity.

## Materials and methods

This randomized cross-over study was conducted in a single center of Montpellier University Hospital. The institutional review board (Comité de Protection des Personnes Sud Méditérannée IV, Montpellier, France) approved the protocol (ref number: 2009-A00087-50). Written informed consent was provided by the patient or surrogates.

### Patients

All consecutive patients with obesity (defined as Body Mass Index (BMI) ≥ 30 kg/m^2^) and without obesity (BMI < 30 kg/m^2^) under invasive mechanical ventilation for at least 24 h were evaluated for inclusion in the study. Patients mechanically ventilated via an endotracheal tube for more than 24 h and ventilated in PSV were included if they have the following inclusion criteria: patient alert and calm corresponding to a Richmond Agitation Sedation Scale (RASS) between − 3 and 0, inspired oxygen inspired fraction (FiO_2_) ≤ 60% with positive end-expiratory pressure (PEEP) ≤ 10 cm H_2_O. Patients were affiliated to the social security system.

The exclusion criteria were age < 18 years, pregnancy, contraindications for an EADi catheter placement, clinical instability for any reason and decision to withhold life-sustaining treatment. Without being completely excluded from the study, some patients may be excluded from secondary analyses due to a lack of data or technical issues.

Patients with obesity were divided into three groups based on BMI for subgroup analysis.

### Ventilator

All the patients included were ventilated using a Servo-I (Maquet Critical Care AB ^®^, Solna, Sweden) equipped with the standard version of the NAVA module. In PSV the assistance was triggered and cycled off by the flow signal and the amount of assistance (pressure support level) is fixed. In NAVA the assistance can be triggered by the EADi signal or by flow signal and the amount of assistance is proportional to electrical activity of the diaphragm (the proportionality factor is set by the physician) [3]. EADi was obtained with a specific nasogastric tube which has ten electrodes at its distal (EADi catheter; Maquet Critical Care, Sölna, Sweden).

### Protocol

After inclusion, the nasogastric tube was replaced by the EADi catheter positioned according to the manufacturer’s recommendations. Each patient was placed in a semi-recumbent position and consecutively ventilated for 30 min with PSV mode and NAVA mode in random order.

In both modes the levels of inspired oxygen fraction (FiO2) and positive end-expiratory pressure (PEEP) were the same as those set before inclusion according to usual practice of the center (Fio_2_ for SpO2 > 92% and PEEP between 5 and 10 cmH2O). The pressure support level and the NAVA level were adjusted by the investigator to obtain a tidal volume (VT) between 6 and 8 ml/kg predicted body weight (PBW) [8]. In the PSV mode flow inspiratory trigger was set to its lowest value without cause autotriggering and cycled off occurred when the inspiratory flow decreased at 30% of its peak inspiratory flow value. In the NAVA mode, neural inspiratory trigger was set at 0.5µV and neural expiratory trigger was set at 70% of peak EADi value. Once ventilator settings realized they were no longer modified during the study period.

### Measurements

Respiratory parameters (airway pressure, flow and EADi) were acquired at 50 Hz from the ventilator via a RS232 cable connected to a computer using commercially available software (Servo-I RCR, version 3.6, Maquet Critical Care) at each mode for 30 min after 5 min of stabilization (Figure S1).

RASS, Behavioral Pain Scale (BPS) and hemodynamics variables (blood pressure and heart rate) were obtained at the beginning and at the end of each mode.

Arterial blood gases were collected at the inclusion and at the end of each mode in patients equipped with an arterial catheter. Respiratory rate (RR), Inspiratory time (Ti), Total respiratory time (Ttot), Tidal Volume (VT), VT/kg (PBW), Minute Ventilation (MV) were obtained from flow recordings; Maximal (Max P_insp_) and mean inspiratory airway pressure (Mean P_insp_) were obtained from airway pressure and maximal EADi from EADi signal. The inspiratory trigger delay (T_d_) was calculated as the time lag between the beginning of EADi swing and the beginning of ventilator inspiratory flow. The inspiratory time in excess (T_iex_, in ms) was calculated as the time lag between the ventilator inspiratory pressurization time and the neural inspiratory time.

The numbers of five types of patient-ventilator dyssynchrony (Ineffective efforts, Auto-triggering, Premature cycling, Delayed cycling, and Double triggering) per minute were determined by the investigator and described in supplemental digital content. In NAVA, double triggering were differentiated into 2 groups: type 1 which was caused by a biphasic EADi signal and type 2 when the cause was different in both modes. Dyssynchronies detection was based on visual inspection of flow, airway pressure and EADi signal. We calculated the Dyssynchrony index (DI) expressed in percentage (dyssynchronous events over the sum of ventilator cycles and ineffective efforts) as previously published [8].

### Statistical analysis

To validate the feasibility of NAVA in patients with obesity, we planned to include up to 10 analyzable patients with obesity, using a convenience sample based on previous studies [8, 12]. We then planned to include up to 10 analyzable patients without obesity to compare the physiological benefits of NAVA between obese and non-obese patients as exploratory outcomes. Data are expressed as median [interquartile range] or mean ± SD according to the type of variable distribution. Kolmogorov-Smirnov test was used to assess normal distribution of data. Comparisons between NAVA and PSV in same patients were performed using paired Student’s t-test or Wilcoxon test according to the data distribution. Comparisons between patients with obesity and patients without obesity were performed using Student’s t-test or Mann Whitney test according to the data distribution. The level of significance was *p* < 0.05.

We evaluated the variability of each ventilatory variables (RR, Ti, Ttot, VT, VM, Max Pinsp, Mean Pinsp, Max EADi) by the coefficients of variation expressed in percentage (standard deviation multiplied by 100 and divided by the mean) as described previously [24].

Linear regression analysis was performed between VT/kg and Max P_insp_.

The statistical analysis was performed using the software MedCalc^®^ (MedCalc Software, Broekstraat, Belgium).

## Results

Ten patients with obesity with a BMI of 35 [31, 32, 33, 34, 35, 36, 37, 38] and 12 without obesity with a BMI at 26 [25, 26, 27, 28] (*p* < 0.01) were included in the study. One patient without obesity was excluded because of the absence of EADi signal (diagnosis of amyotrophic lateral sclerosis), thus 21 patients were analyzed (Figure S2) with a total of 1790 ± 873 respiratory cycles analyzed per patient. Admission pathology in Intensive Care Unit (ICU) and clinical characteristics of the 21 patients who concluded the study are summarized in Table 1. No patients had medical history of chronic obstructive pulmonary disease and none was tracheotomized. Arterial blood gases were available for 18 patients. At the inclusion we found no significant difference between patients with and without obesity for PaO_2_/FiO_2_ (204 [188–258] vs. 195 [178–300] mmHg), pH and PaCO_2_ (Table S1). No patient suffered from hypercapnia above 50 mmHg.

**Table 1:** Main characteristics of the 10 patients with obesity and the 11 patients without obesity included

Patient	Obesity	Age (Yr)	Gender	Post-operative	Main diagnosis	Height (cm)	Weight (kg)	BMI (kg/m^2^)	SAPS II	Time between		Total duration of invasive ventilation (days)	Outcome
										intubation and inclusion (days)	inclusion and weaning (days)		
With obesity	100%	65 [61–73]	20% F	70% Yes	-	171 [168–173]	100 [92–111]	35 [31–38]	60 [43–75]	3[2–5]	4[1–19]	10[3–23]	40% Died
1	Yes	63	M	Yes	Bowel obstruction	170	103	36	63	8	3	11	Survived
2	Yes	60	M	Yes	subdural empyema	173	91	31	57	3	11	14	Survived
3	Yes	66	M	Yes	hepatectomy	172	93	31	22	3	183	186	Died
4	Yes	48	M	Yes	Acute pancreatitis	172	113	38	39	2	0	2	Died
5	Yes	79	F	No	coma	155	76	32	77	1	1	2	Died
7	Yes	75	M	Yes	Peritonitis	176	124	40	70	1	1	2	Survived
9	Yes	68	M	Yes	acute cholecystitis	170	90	31	85	5	21	26	Died
16	Yes	35	M	No	Pneumonia	167	106	38	34	2	51	53	Survived
18	Yes	82	F	No	Stroke	157	97	39	76	8	1	9	Survived
20	Yes	62	M	Yes	Peritonitis	180	112	35	55	2	4	6	Survived
Without obesity	0%	69 [56–77]	45% F	45% Yes	-	165 [163–174]	68 [68–79]	26 [25–28]	48 [39–59]	2[1–6]	2[1–5]	4[3–9]	18% Died
6	No	51	F	Yes	Stroke	183	94	28	54	18	14	32	Survived
8	No	61	F	Yes	Acute pancreatitis	165	68	25	48	1	3	4	Survived
10	No	62	M	Yes	abdominal trauma	178	86	27	41	6	1	7	Survived
11	No	82	F	Yes	Subdural hematoma	160	67	26	42	3	0	3	Survived
12	No	78	F	No	Cardiac arrest	155	67	28	91	5	10	15	Died
13	No	79	M	No	Stroke	180	76	23	35	2	1	3	Died
14	No	69	M	No	Hepatic Coma	163	73	27	59	1	1	2	Survived
15	No	71	M	No	Pneumonia	170	82	28	60	2	2	4	Survived
17	No	50	M	No	Status epilepticus	163	68	26	37	1	2	3	Survived
19	No	76	F	No	Pneumonia	165	68	25	58	1	6	7	Survived
21	No	46	M	Yes	Liver injury	170	68	24	33	11	0	11	Survived
*P*	< 0.01	0.75	0.36	0.39		0.57	< 0.01	< 0.01	0.38	0.85	0.28	0.48	0.36

Summary data are presented as median [interquartile range]. *P*: *P* value calculated between patients with and without obesity using Mann Whitney test. BMI: Body Mass Index; SAPS II: Simplified Acute Physiology Score II

All patients concluded the study and none had suffered from adverse events in both groups. Ventilatory settings in NAVA and in PSV are shown in Table S2. In PSV mode, PSV level exceeded 10 cmH_2_O in 1 patient with obesity and 3 without obesity. In NAVA mode, NAVA level was never higher than 2 cmH_2_O/µV. The monitored ventilatory variables and parameters are presented in Table S3. Patients with obesity tend to have lower VT with NAVA versus PSV, respectively 6.7 mL/kg predicted body weight [5.9–7.1] versus 7.2 mL/kg [6.2–8.2], *p* < 0.05. In patients without obesity this difference was not significant. Minute ventilation was similar between NAVA and PSV. There was no significant difference in Max EADi between patients with or without obesity. In PSV mode it was 6.7 [4.1–11] µV in patient with obesity versus 8.8 [4.6–11.1] µV in patients without obesity (*p* = 0.38), and in NAVA mode it was 7 [4.5–10.4] versus 8.9 [4.9–9.5] (*p* = 0.627). Compared to PSV, NAVA was significantly associated with more respiratory cycles with Max P_insp_ >30 cmH_2_O (Figure S3) and with VT < 5 ml/kg PBW (Table S4).

**Fig. 1:**
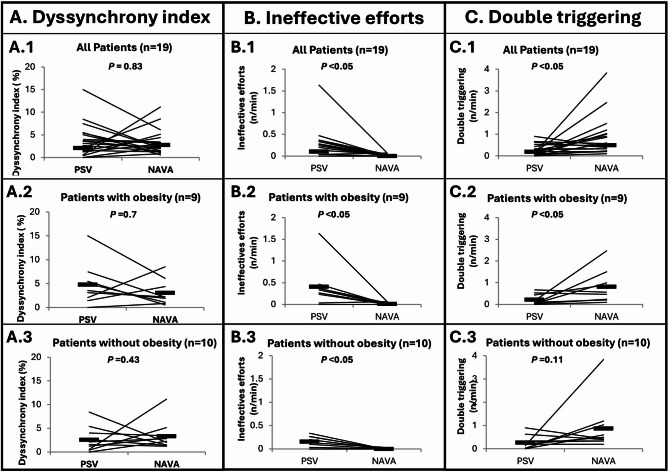
Individual variation of the Dyssynchrony index (DI) for 19 of 21 patients, for 9 of 10 patients with obesity (*Left*) and for 10 of 11 patients without obesity (*right*) during Pressure Support Ventilation (PSV) and Neurally Adjusted Ventilatory Assist (NAVA). The horizontal bars represent the mean values

In patients with obesity, NAVA versus PSV was associated with a reduction in T_d_ (0 [0–5] vs. 106 [34–125] ms, *p* < 0.05) and T_iex_ (96 [94–102] vs. 145 [137–202] ms, *p* < 0.01). Patient-ventilator Dyssynchronies are reported in Table 2. A technical issue occurred in recording of respiratory cycles in 2 patients, thus 19 patients were analyzed for this part of the study. Despite the decrease in ineffective efforts (0 [0-0.03] vs. 0.33 [0.23–0.37] events/min, *p* < 0.05), the prevalence of total dyssynchronies was similar in NAVA and in PSV (2.2% [1.1–4.4] vs. 3.7% [2.4–5.6], *p* = 0.68). Indeed, the NAVA ventilation was associated with an increase in Type 1 double triggering. Figure 1 shows individual DI in NAVA and in PSV. DI exceeded 10% in patients without obesity in NAVA and in one patient with obesity in PSV. There was no significant difference between PSV and NAVA. Subgroup analysis by BMI showed no consistent trend or statistically significant differences between PSV and NAVA across BMI groups (Table S5).

**Table 2:** Total and specific dyssynchrony events for PSV and NAVA in all patients, with and without obesity

	All patients (*n* = 19)			Patients with obesity (*n* = 9)			Patients without obesity (*n* = 10)		
	PSV	NAVA	*P*	PSV	NAVA	*P*	PSV	NAVA	*P*
T_d_ (ms)	**113 [34–152]**	**0 [0–10]**	**< 0.01**	**106 [34–125]**	**0 [0–5]**	**0.02**	**86 [9-156]**	**0 [0–12]**	**0.02**
T_iex_ (ms)	**179 [124–218]**	**105 [97–110]**	**< 0.01**	**145 [137–202]**	**96 [94–102]**	**< 0.01**	**192 [133–249]**	**110 [107–117]**	**0.03**
Total dyssynchronies (n/min)	0.6 [0.3–1.2]	0.6 [0.5–1.1]	0.71	0.9 [0.5–1.3]	0.6 [0.3-1]	1	0.5 [0.2–0.9]	0.6 [0.5–1.1]	0.63
DI (%)	3.1 [1.1–4.7]	2.2 [1.4–3.9]	0.77	3.7 [2.4–5.6]	2.2 [1.1–4.4]	0.68	1.7 [0.8–3.7]	2.6 [1.6–3.3]	0.37
Ineffective efforts (n/min)	**0.27 [0.07–0.33]**	**0 [0–0]**	**< 0.01**	**0.33 [0.23–0.37]**	**0 [0-0.03]**	**0.02**	**0.17 [0.05–0.27]**	**0 [0–0]**	**0.02**
Double triggering (n/min)	**0.17 [0.03–0.38]**	**0.5 [0.33–1.02]**	**0.01**	**0.1 [0.03–0.43]**	**0.57 [0.23-1]**	**0.02**	0.2 [0.06–0.33]	0.47 [0.35–0.93]	0.09
Double triggering type 1 (n/min)	**0 [0–0]**	**0.37 [0.18–0.7]**	**< 0.01**	**0 [0–0]**	**0.4 [0.2–0.87]**	**< 0.01**	**0 [0–0]**	**0.33 [0.18–0.51]**	**< 0.01**
Double triggering type 2 (n/min)	0.17 [0.03–0.38]	0.13 [0.05–0.2]	0.34	0.1 [0.03–0.43]	0.1 [0.03–0.2]	0.74	0.2 [0.06–0.33]	0.13 [0.07–0.19]	0.31
Auto triggering (n/min)	0.07 [0-0.13]	0 [0-0.07]	0.11	0.1 [0.07–0.17]	0.03 [0-0.1]	0.31	0.03 [0-0.11]	0 [0-0.03]	0.14
Premature cycling (n/min)	**0.03 [0-0.07]**	**0 [0–0]**	**0.03**	0.07 [0-0.13]	0 [0–0]	0.07	0.02 [0-0.06]	0 [0–0]	0.16
Late cycling (n/min)	0.03 [0-0.07]	0 [0-0.03]	0.12	0.03 [0-0.07]	0 [0-0.03]	0.40	0.02 [0-0.06]	0 [0–0]	0.20

Data are presented as median [interquartile range]. *P*: *P* value calculated between PSV and NAVA using Wilcoxon test. PSV = Pressure Support Ventilation; NAVA = Neurally Adjusted Ventilatory Assist; DI = Dyssynchrony index n/min = Number of events/min; T_d_ inspiratory trigger delay; T_iex_ inspiratory time in excess

**Fig. 2:**
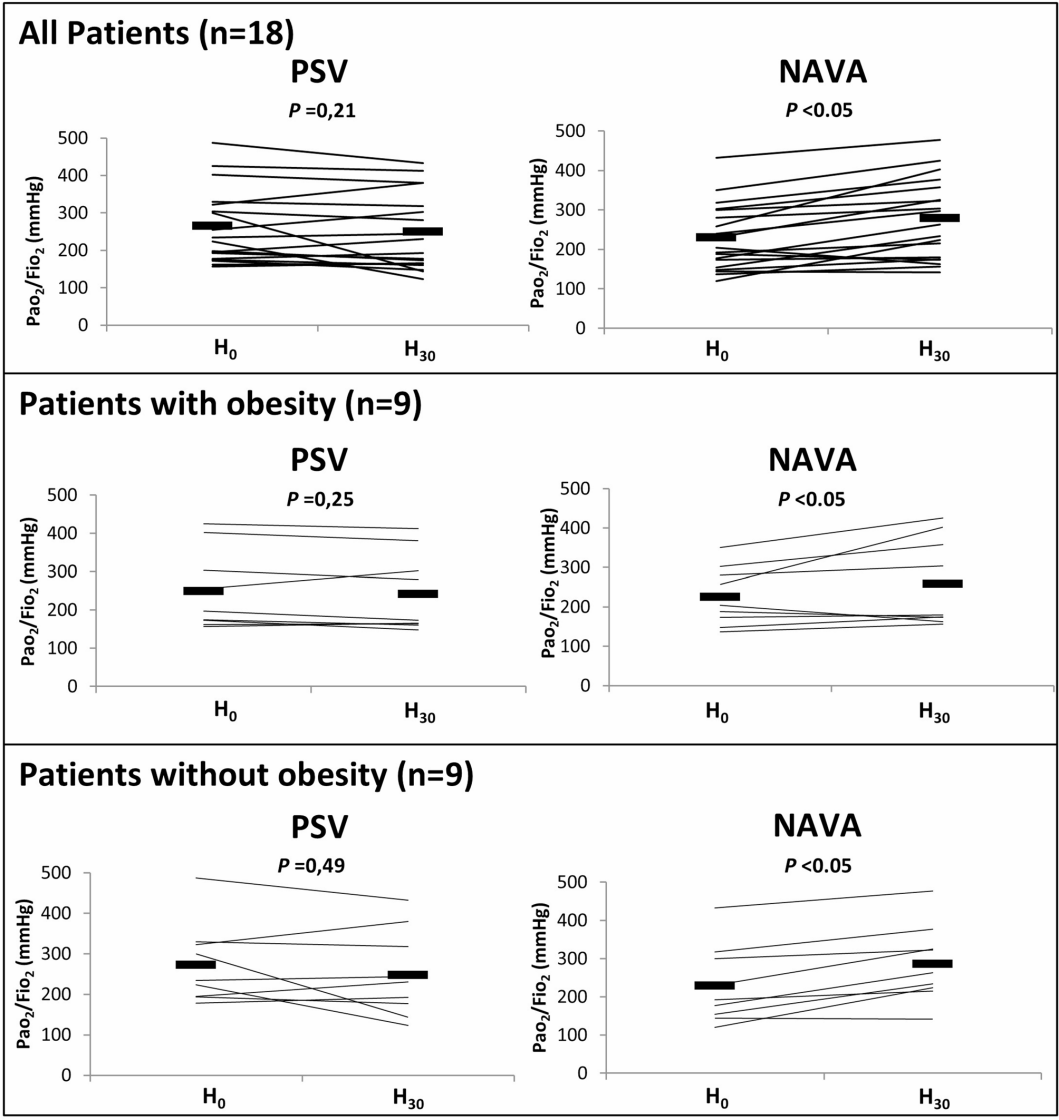
Individual variations in PaO_2_/FiO_2_ ratio for 18 of 21 patients (*top*), for 9 of 10 patients with obesity (*middle*) and for 9 of 11 patients without obesity (*bottom*) after mechanical ventilation with Pressure Support Ventilation (PSV, *left*) and with Neurally Adjusted Ventilatory Assist (NAVA, *right*). The horizontal bars represent the mean values. Pao_2_ = partial pressure of arterial oxygen; Fio_2_ = inspired oxygen fraction; H 0 = baseline; H 30 = after 30 min of mechanical ventilation; * *P* < 0.05 between H 0 and H 30

Compared with baseline, the PaO_2_/FiO_2_ ratio was significantly higher at the end of NAVA ventilation but not at the end of PSV ventilation Fig. 2. No significant association was found between PaO_2_/FiO_2_ and variability of VT or Max P_insp_ (Figure S4). Arterial pH, PaCO_2_, PaO_2_/FiO_2_ were not significantly different between patients with and without obesity. Over the study period, Hemodynamics parameters, RASS and BPS scores were not significantly different between the two modes. The obesity factor was never significant.

**Fig. 3:**
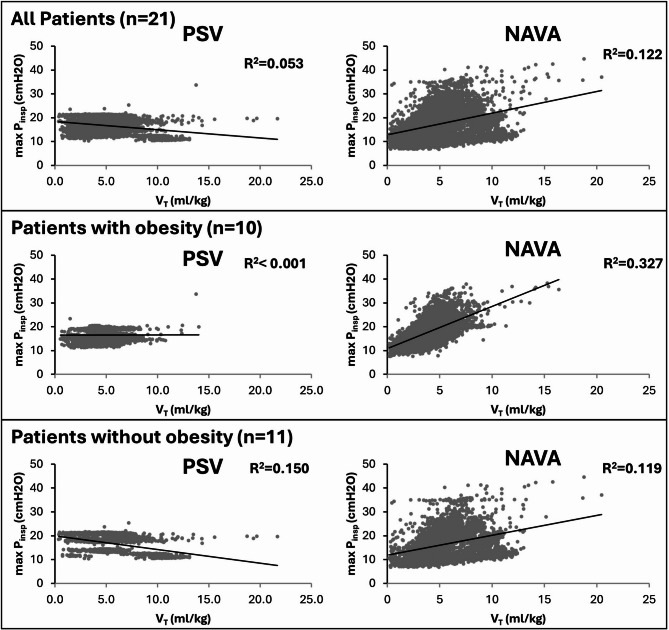
Scatter plots of Maximal inspiratory airway pressure (Max P_insp_, cmH2O) vs. tidal volume (VT, mL/kg) measured in all patients (*top*), with obesity (*middle*) and without obesity (*bottom*) during Pressure Support Ventilation (PSV, *left*) and Neurally Adjusted Ventilatory Assist (NAVA, *right*). Lines represent linear regression lines

Figure 3 represents the relationship between VT and Max P_insp_ in NAVA and in PSV, in patients with and without obesity. Max P_insp_ remained essentially constant in PSV mode across a range of VT, suggesting limited adaptation of support to the patient’s effort. By contrast, in NAVA, Max P_insp_ scaled with VT, showing more variability. Figures S5–S8 display individual patient–level pressure–volume relationships. Variability of all ventilatory parameters (RR, VT, Max P_insp_) are presented in Table S6. Ventilatory variability was significantly higher with NAVA, with a mean inspiratory airway pressure of 15 [10, 11, 12, 13, 14, 15, 16, 17, 18, 19, 20, 21] % versus 3 [2, 3, 4] % in overall patients, 16% [11, 12, 13, 14, 15, 16, 17, 18, 19, 20, 21] versus 4% [2, 3, 4] in patients with obesity, 12 [9, 10, 11, 12, 13, 14, 15, 16, 17, 18, 19, 20] % versus 3 [1, 2, 3] % in patients without obesity.

## Discussion

This study showed that NAVA ventilation was feasible and safe in critically ill patients with obesity, allowing a reduction in ineffective effort, an improvement in oxygenation and an increase of variability compared to PSV ventilation.

To our knowledge this is the first study to report the use of NAVA in the specific group of patients with obesity. The collection of the EADi signal has always been possible, and no patient was excluded in this group which confirms the feasibility and safety of NAVA in patients with obesity. Obesity does not appear to cause difficulty for the utilization of NAVA. In patients without obesity, only one was excluded because the EADi signal necessary for NAVA was absent. This lack of signal highlighted a diaphragmatic paralysis related to amyotrophic lateral sclerosis whose diagnosis was made posteriori. Diaphragmatic dysfunction is a known limitation of NAVA [25] as the neural stimulus of the diaphragm is used to trigger the ventilatory and to determine the level of pressure support provided.

NAVA ventilation was associated with a significant reduction in ineffective efforts in both groups of patients. Ineffective effort occurs when patient effort is insufficient to overcome the burden of intrinsic PEEP. While in PSV patients must overcome the intrinsic PEEP before triggering an inspiration, there is no possible influence of intrinsic PEEP in NAVA on the beginning of the cycle, since assistance begins with the patient’s neural effort. Using a neural trigger allows to eliminate the link between hyperinflation and patient-ventilator dyssynchrony [3]. Moreover, in NAVA the inspiratory assistance is proportional to patient’s effort which limiting the risk of over or under assistance, harmful to the diaphragm [26]. High pressure level leads to an increasing of tidal volume (which promotes intrinsic PEEP) and an inhibition of central control (which reduces the intensity of effort) which is at the origin of ineffective efforts [27].

Despite the reduction of ineffective effort with NAVA the dyssynchronies index was similar in NAVA and in PSV in contrast to previous reports [5, 12, 13, 28]. In these studies the authors found dysynchronies index between 12 and 40.2% in PSV and between 4, 5 to 10.8% in NAVA and the difference is always significantly in favor of NAVA. Compared to these studies, the incidence of patient ventilator dyssynchrony is very low in our work which may explain the absence of significant difference between NAVA and PSV. The lower incidence of patient ventilator dyssynchronies in PSV in our study could have 3 explanations. The first is the absence of a patient with severe obstructive disease in our work in contrast to Schmidt et al., and Piquilloud et al. studies. Due to a higher intrinsic PEEP these patients are more likely to have a high incidence of dyssynchrony [29]. Second, we set the pressure support level lower than previous studies which limit the risk of over assistance. High level of assistance leads to an increasing of tidal volume (which promotes intrinsic PEEP) and an inhibition of central control (which reduces the intensity of effort) and causes patients ventilator dyssynchronies [27]. In this study, low level of pressure assistance probably could explain why incidence of patient-ventilator dyssynchronies are similar in NAVA and in PSV. Despite these low levels of pressure support, no patients suffer from respiratory clinical signs of self-inflicted lung injury (SILI) [30] and/or acidosis which confirms that our settings were adapted to the patients. Finally, Schmidt et al., Bertrand et al. and Piquilloud et al. studies compared NAVA and PSV in non-invasive ventilation and patient-ventilator dyssynchronies can be increased by leaks through the facemask [31].

As previously reported, there was more double triggering type 1 in NAVA [4, 5, 6, 13]. This increase is explained for technical reasons. As well described by Piquilloud et al. [5], the filtered Eadi signal used by NAVA to command the ventilator sometimes has a biphasic aspect. In NAVA, both triggering and cycling are driven by the EAdi. A fall in EAdi amplitude after the first peak is interpreted as end-inspiration, prompting cycling-off and termination of pressurization. A subsequent rise in EAdi (second peak) is then interpreted as the onset of a new inspiratory effort, leading the ventilator to deliver a second pressurization. This produces a NAVA-specific form of double triggering. Accordingly, we distinguish type 1 “technical” double triggering inherent to NAVA, and type 2 “standard” double triggering attributable to other causes. Type 1 double triggering is difficult to mitigate. Lowering trigger sensitivity may be deleterious with ineffective efforts and increased patient effort. Increasing the NAVA level can reduce effort and attenuate EADi amplitude, thereby limiting the double peak. Type 2 double triggering management first seeks external contributors and then optimizes trigger sensitivity and the NAVA level.

In this study we found an improvement in oxygenation in NAVA compared to PSV. This improvement of oxygenation in NAVA is reported for long duration of NAVA [8] but not for short length. Several hypotheses could explain this improvement of oxygenation in NAVA. Mechanically ventilated patients with obesity have an increased risk of atelectasis and hypoxemia [17] and recruitment maneuver reduce the atelectasis and improved oxygenation [32]. The presence of more pneumatic cycles with Max P_insp_ >30 cmH_2_O in NAVA could be assimilated to alveolar auto-recruitment and could explain a better oxygenation. Moreover, compared to PSV there was no more breathing cycle with an excessive VT and NAVA should not increase the risk of ventilation induced injury. Better oxygenation could also be explain by an increasing of respiratory variability [33, 34] and the diminution of ineffective triggering in NAVA.

Obesity is characterized by reduced functional residual capacity, decreased chest-wall compliance, higher pleural/abdominal pressures, and greater atelectasis susceptibility, often necessitating higher PEEP and inspiratory pressures [17]. In this context, NAVA’s effort-proportional assistance helps adapt pressurization to patient demand reducing lung collapse. NAVA also improves patient–ventilator synchrony, preserves physiologic variability, and limits both over- and under-assistance, which are associated with lung and diaphragm injury [26]. NAVA in patients with obesity was also associated with lower VT and higher respiratory rates than PSV with similar MV. Physiological studies found that, at baseline, obese subjects have higher RR and lower VT compared to non-obese subjects [35]. During an increase in ventilatory demand (for example during an effort), these patients preferentially increase their respiratory rate and decrease their tidal volume [22]. Thus, the modification of breathing pattern in NAVA could correspond to a more physiological ventilation in patients with obesity. Difference in maximal EAdi between patients with and without obesity was not statistically significant. Although it could be thought that the EAdi signal intensity reflects a greater esophagus–diaphragm distance (as hypothesized in patients with obesity), it is in fact mainly influenced by the strength of the patient’s inspiratory effort.

In overall patients we showed a Max P_insp_ remained essentially constant in PSV mode across a range of VT, suggesting limited adaptation of support to the patient’s effort. Achieving higher ventilation therefore required greater patient effort. By contrast, in NAVA, Max P_insp_ scaled with VT, showing physiologically appropriate variability and indicating better coupling between neural drive and delivered pressure. Previous studies suggest the clinical benefit of increased breathing variability which was associated with successful ventilatory weaning [36, 37], lower duration of mechanical ventilation [38] and higher survival rate [38, 39].

There are several limitations to this work. First, although the crossover design strengthens the validity of the results, the relatively small sample size limits the generalizability of the study. Second, the duration of ventilation in our study was probably too short to evaluate the long term safety of NAVA ventilation patients with obesity. As it is a physiological study, the effect of the NAVA on morbidity and prognosis for patients with obesity has not been studied. Third, our group of patients with obesity was mostly comprised of moderate to severe obesity (BMI = 35 kg/m^2^ median). Further studies are necessary to evaluate NAVA in a population of morbidly obese (BMI > 40 kg/m^2^). Similarly, patients without obesity were nonetheless overweight, with a median BMI of 26 kg/m². This reflects consecutive enrollment, participants were not selected on the basis of BMI. Fourth, the higher incidence of ineffective efforts during PSV may be attributed to an excessively high flow trigger [27]. However, the trigger sensitivity was set to a very low (i.e., highly sensitive) value, which could conversely increase the risk of self-triggering.

Further studies in larger cohorts including morbidly obese patients, with longer follow-up, are needed to confirm the clinical benefits of NAVA (improved trigger precision, enhanced ventilatory variability, and better oxygenation) on outcomes such as weaning success, ventilation duration and mortality in patients with obesity.

## Conclusion

This study reports for the first time the feasibility and the safety of NAVA in patients with obesity. The NAVA has reduced the ineffective efforts and improved oxygenation in patients with obesity compared to PSV. Subsequent studies on the interest of NAVA on the duration of weaning and respiratory prognosis of patients with obesity could be considered.

## Supplementary Information

Below is the link to the electronic supplementary material.

Supplementary Material 1

## Data Availability

The datasets used and/or analysed during the current study are available from the corresponding author on reasonable request.

## Abbreviations

BMI: Body Mass Index
BPS: Behavioral Pain Scale
DI: Dyssynchrony Index
EADi: Electrical Activity of the Diaphragm
EMG: Electromyogram
FiO2: Inspired Oxygen Fraction
ICU: Intensive Care Unit
MV: Minute Ventilation
NAVA: Neurally Adjusted Ventilatory Assist
PaCO2: Arterial Partial Pressure of Carbon Dioxide
PaO2: Arterial Partial Pressure of Oxygen
PBW: Predicted Body Weight
PEEP: Positive End Expiratory Pressure
Pins: Inspiratory Pressure
PSV: Pressure Support Ventilation
RASS: Richmond Agitation Sedation Scale
RR: Respiratory Rate
SAPS II: Simplified Acute Physiology Score II
Td: Trigger delay
Ti: Inspiratory Time
Tiex: Time in excess
Ttot: Totale Respiratory Time
VT: Tidal Volume
