# Supplementary material for: Neurally adjusted ventilatory assist in critical care patients with and without obesity: a prospective randomized crossover study
Source: Ann Intensive Care. 2025 Aug 29;15:128. doi: 10.1186/s13613-025-01552-x (PMC12394103; doi:10.1186/s13613-025-01552-x)

# SUPPLEMENTARY MATERIAL

**Neurally adjusted ventilatory assist in critical care patients with and without obesity: a prospective randomized crossover study**

Matthieu Conseil, Samir Jaber, Fabrice Galia, Nicolas Molinari, Gerald Chanques, Audrey De Jong, Mathieu Capdevila

**Table of contents**

- **Additional method**
- **Table S1:** Arterial blood gases before inclusion
- **Table S2**: Ventilator settings obtained at the baseline of each ventilatory period for PSV and NAVA in all patients, with and without obesity.
- **Table S3**: Main monitored parameters obtained during each ventilatory period for PSV and NAVA in all patients, with and without obesity.
- **Table S4**: Percentage of time spent in different range of VT for PSV and NAVA in all patients, with and without obesity.
- **Table S5:** Total and specific dyssynchrony events for PSV and NAVA in patients with obesity, according to three BMI groups.
- **Table S6**: Variability of ventilatory parameters during each ventilatory period for PSV and NAVA in all patients, with and without obesity.
- **Figure S1**: Representative tracing of airway pressure, flow, respiratory volume and electrical activity of the diaphragm (EADi) during (A) pressure support ventilation (PSV) and (B) neurally adjusted ventilatory assist (NAVA).
- **Figure S2**: Flow chart
- **Figure S3**: Individual percentage of pneumatic cycle with maximal inspiratory airway pressure higher than 30 cmH2O during Pressure Support Ventilation (PSV) and Neurally Adjusted Ventilatory Assist (NAVA) in all patients, with and without obesity. Plots represent individual percentages. The horizontal bars represent the mean values in each group.
- **Figure S4**: Scatter plots of the variation of PaO2/FiO2 ratio according to tidal volume variability (VT variability) (left) or Maximal inspiratory airway pressure variability (Max P_insp_ variability) (right) measured in all patients (top), with obesity (middle) and without obesity (bottom) during pressure support ventilation (PSV) and neurally adjusted ventilatory assist (NAVA).
- **Figure S5**: Individualized scatter plots of maximal inspiratory airway pressure (Pmax, cmH2O) vs. tidal volume (Vt, mL/kg) measured in patients with obesity during Pressure Support Ventilation. Lines represent linear regression lines.
- **Figure S6**: Individualized scatter plots of maximal inspiratory airway pressure (Pmax, cmH2O) vs. tidal volume (Vt, mL/kg) measured in patients with obesity during Neurally Adjusted Ventilatory Assist. Lines represent linear regression lines.
- **Figure S7**: Individualized scatter plots of maximal inspiratory airway pressure (Pmax, cmH2O) vs. tidal volume (Vt, mL/kg) measured in patients without obesity during Pressure Support Ventilation. Lines represent linear regression lines.
- **Figure S8**: Individualized scatter plots of maximal inspiratory airway pressure (Pmax, cmH2O) vs. tidal volume (Vt, mL/kg) measured in patients without obesity during Neurally Adjusted Ventilatory Assist. Lines represent linear regression lines.

**Additional Method**

**Definitions of patient ventilator Dyssynchronies**

We detected five types of Dyssynchronies by visual inspection by the investigators of Flow, Pressure and EADi Curve. Samples of dyssynchronies are shown in Figure 1. The definitions of dyssynchronies were as follows:

- Ineffective effort: presence of a characteristic inspiratory neural activity (EADi) not followed by a ventilator-delivered pressurization.

- Auto-triggering: presence of a ventilator delivered pressurization without an inspiratory neural activity.

- Double triggering: presence of 2 ventilator-delivered pressurization separated by an expiratory twice shorter than the mean patient's neural inspiratory time. Double triggering were differentiated into 2 groups : type 1 when double triggering is caused by a biphasic EADi signal and Type 2 when double triggering is provoked by any other cause.

- Premature cycling: duration of pressurization delivered by the ventilator is twice shorter than the patient's neural inspiratory time.

- Delayed cycling: duration of pressurization delivered by the ventilator is twice longer than the patient's neural inspiratory time.

**Table S1**: Arterial blood gases before inclusion

|  | All patients  (n=18) | Patients with obesity  (n=9) | Non obeses patients (n=9) | p |
| --- | --- | --- | --- | --- |
| pH | 7.43 [7.38-7.5] | 7.43 [7.38-7.47] | 7.45 [7.39-7.51] | ns |
| PaCO2 (mmHg) | 36 [32-38] | 36 [34-38] | 36 [31-39] | ns |
| PaO2 (mmHg) | 98 [79-119] | 94 [84-102] | 116 [77-132] | ns |
| HCO3- (mmol/L) | 24 [22-26] | 24 [22-25] | 24 [24-26] | ns |
| SaO2 (%) | 98 [96-99] | 97 [96-98] | 98 [96-99] | ns |
| PaO2/Fio_2_ (mmHg) | 201 [180-295] | 204 [188-258] | 195 [178-300] | ns |
| Data are presented as median [interquartile range]. p: p value calculated between patients with and without obesity using Mann Whitney test. PaO2: partial pressure of arterial oxygen; PaCO2 = partial pressure of arterial carbon dioxide; HCO3- = plasma bicarbonate; SaO2 = arterial saturation in oxygen; Fio_2_: oxygen inspired fraction; ns= not significant. | | | | |

**Table S2**: Ventilator settings obtained at the baseline of each ventilatory period for PSV and NAVA in all patients, with and without obesity.

|  | All patients (n=21) | | Patients with obesity  (n=10) | | Patients without obesity (n=11) | |
| --- | --- | --- | --- | --- | --- | --- |
|  | PSV | NAVA | PSV | NAVA | PSV | NAVA |
| Pressure support level (cmH2O) | 9±3 | NA | 8±3 | NA | 9±2 | NA |
| NAVA gain level (cmH2O/µv) | NA | 1,2±0,4 | NA | 1,2±0,4 | NA | 1,3±0,4 |
| Flow inspiratory trigger (L/min) | 1 ± 0 | 1 ± 0 | 1 ± 0 | 1 ± 0 | 1 ± 0 | 1 ± 0 |
| Neural inspiratory trigger (µV) | NA | 0,5±0 | NA | 0,5±0 | NA | 0,5±0 |
| Flow expiratory trigger (% of max peak flow) | 30 ± 0 | 30 ± 0 | 30 ± 0 | 30 ± 0 | 30 ± 0 | 30 ± 0 |
| NAVA expiratory trigger (% of max EADI) | NA | 70±0 | NA | 70±0 | NA | 70±0 |
| Inspiratory rise (ms) | 150 ± 0 | NA | 150 ± 0 | NA | 150 ± 0 | NA |
| PEEP (cmH2O) | 7±2 | 7±2 | 7±2 | 7±2 | 6±2 | 6±2 |
| Fio_2_ (%) | 44±9 | 44±9 | 43±9 | 43±9 | 45±8 | 45±8 |
| Data are presented as mean ± SD. PSV = Pressure Support Ventilation; NAVA = Neurally Adjusted Ventilatory Assist; EADi = Diaphragm Electrical Activity; PEEP = positive end-expiratory pressure; Fio_2_ = Oxygen Inspiratory Fraction. There was no significant difference between patients with and without obesity using Student t-test. | | | | | | |

**Table S3**: Main monitored parameters obtained during each ventilatory period for PSV and NAVA in all patients, with and without obesity.

|  | All patients (n=21) | | | Patients with obesity (n=10) | | | Patients without obesity (n=11) | | |
| --- | --- | --- | --- | --- | --- | --- | --- | --- | --- |
|  | PSV | NAVA | P | PSV | NAVA | P | PSV | NAVA | P |
| RR (breaths/min) | **24 [18-32]** | **26 [23-37]** | **0.004** | **24 [20-30]** | **25 [23-35]** | **0.019** | 25 [18-34] | 29 [22-38] | 0.067 |
| Ti (s) | 0.8 [0.7-1] | 0.8 [0.6-1.1] | 0.812 | 0.8 [0.7-0.9] | 0.81 [0.6-0.9] | 1 | 0.8 [0.7-1] | 0.9 [0.7-1.1] | 0.921 |
| Ttot (s) | **2.6 [1.9-3.7]** | **2.5 [1.8-3.3]** | **0.010** | 2.7 [2.1-3.2] | 2.6 [1.9-3.2] | 0.084 | 2.6 [1.7-4.1] | 2.5 [1.7-3.3] | 0.084 |
| VT (mL) | **442 [364-543]** | **422 [344-491]** | **0.006** | **475 [412-547]** | **445 [393-487]** | **0.019** | 408 [354-476] | 370 [333-460] | 0.147 |
| VT (mL/kg) | **6.6 [6-7.5]** | **6.3 [5.8-7.1]** | **0.006** | **7.2 [6.2-8.2]** | **6.7 [5.9-7.1]** | **0.019** | 6.5 [5.8-7] | 6.1 [5.8-7] | 0.174 |
| MV (L/min) | 11 [8.6-13.1] | 10.9 [9.1-12.5] | 0.192 | 10.3 [8.9-11.5] | 10.1 [9.2-12.1] | 0.556 | 11 [8.5-13.3] | 12 [8.5-12.7] | 0.320 |
| Max P_insp_ (cmH2O) | **16 [14-18]** | **18 [16-20]** | **0.045** | **16 [15-18]** | **18 [17-20]** | **0.005** | 18 [13-18] | 17 [13-19] | 0.702 |
| Mean P_insp_ (cmH2O) | **13 [11-15]** | **12 [10-14]** | **0.005** | 12 [12-14] | 12 [11-14] | 0.105 | **14 [11-15]** | **11 [9-14]** | **0.032** |
| Max EADi (µV) | 8.3 [4.6-11.3] | 8 [5-10.3] | 0.57 | 6.7 [4.1-11] | 7 [4.5-10.4] | 1 | 8.8 [4.6-11.1] | 8.9 [4.9-9.5] | 0.577 |
| Data are presented as median [interquartile range]. P: P value calculated between PSV and NAVA using Wilcoxon test. PSV = Pressure Support Ventilation; NAVA = Neurally Adjusted Ventilatory Assist; RR = Respiratory Rate; Ti = Inspiratory Time; Ttot = Total time of respiratory cycle; VT = Tidal Volume; VM = Minute Ventilation ; Max P_insp_ = Maximal inspiratory airway pressure; Mean P_insp_ = Mean inspiratory airway pressure; Max EADi: Maximal electrical activity of the diaphragm | | | | | | | | | |

**Table S4**: Percentage of time spent in different range of VT for PSV and NAVA in all patients, with and without obesity.

|  | All patients (n=21) | | | Patients with obesity (n=10) | | | Non obese patients (n=11) | | |
| --- | --- | --- | --- | --- | --- | --- | --- | --- | --- |
|  | PSV | NAVA | P | PSV | NAVA | P | PSV | NAVA | P |
| VT < 5 mL/kg | **7.5 [2.5-15.7]** | **18.1 [9.5-31.5]** | **0.04** | 9 [3.5-17.9] | 20.3 [7.1-29.8] | 0.23 | 5.4 [2.1-13.3] | 17.3 [9.6-24.9] | 0.08 |
| VT = 5-12 mL/kg | 90.8 [80.5-96.2] | 78.7 [65.8-87.1] | 0.29 | 90.8 [81-94.2] | 77 [66.4-92.9] | 0.23 | 88.2 [67.1-97.1] | 81.5 [63.5-83.6] | 0.77 |
| VT > 12 mL/kg | 0.5 [0-1.4] | 0.3 [0-1] | 0.67 | 0.5 [0-1.1] | 0.7 [0-1] | 0.55 | 0.6 [0-1.6] | 0.3 [0.2-1] | 0.31 |
| Data are presented as median [interquartile range]. P: P value calculated between PSV and NAVA using Wilcoxon test. PSV = Pressure Support Ventilation; NAVA = Neurally Adjusted Ventilatory Assist; VT= Tidal Volume. | | | | | | | | | |

**Table S5**: Total and specific dyssynchrony events for PSV and NAVA in patients with obesity, according to three BMI groups.

|  | Group 1 (BMI 30.4-31.4, n=3) | | | Group 2 (BMI 31.6-38.0, n=3) | | | Group 3 (BMI 38.2-40.0, n=3) | | | |
| --- | --- | --- | --- | --- | --- | --- | --- | --- | --- | --- |
|  | PSV | NAVA | P | PSV | NAVA | P | PSV | NAVA | P | |
| Total dyssynchronies (n/min) | 0.9 [0.9-2.0] | 0.6 [0.1-2.7] | 0.75 | 1.0 [0.0-1.3] | 0.5 [0.2-1.4] | 1.00 | 0.5 [0.3-0.6] | 1.0 [0.2-1.8] | 0.50 | |
| DI (%) | 3.7 [3.5-5.2] | 2.3 [0.5-5.9] | 0.50 | 5.2 [0.0-6.3] | 2.1 [0.6-7.2] | 1.00 | 2.0 [1.3-3.1] | 1.0 [0.2-1.8] | 0.75 | |
| Ineffective efforts (n/min) | **0.5 [0.4-1.6]** | **0.1 [0.0-0.1]** | 0.25 | **0.2 [0.0-0.5]** | **0.0 [0.0-0.0]** | 0.18 | **0.2 [0.0-0.3]** | **0.0 [0.0-0.1]** | 0.50 | |
| Double triggering (n/min) | 0.1 [0.0-0.2] | 0.3 [0.0-2.5] | 0.18 | **0.4 [0.0-0.6]** | **0.5 [0.1-1.4]** | 0.75 | **0.0 [0.0-0.1]** | **1.0 [0.2-1.5]** | 0.25 | |
| Double triggering type 1 (n/min) | **0.5 [0.0-1.6]** | **0.1 [0.0-2.2]** | 0.65 | **0.4 [0.0-0.6]** | **0.4 [0.1-1.4]** | 0.75 | **0.0 [0.0-0.1]** | **0.3 [0.2-1.4]** | 0.25 | |
| Double triggering type 2 (n/min) | 0.1 [0.0-0.2] | 0.2 [0.0-0.2] | 0.18 | 0.0 [0.0-0.0] | 0.0 [0.0-0.1] | 0.32 | 0.0 [0.0-0.0] | 0.1 [0.0-0.7] | 0.180 | |
| Auto triggering (n/min) | 0.1 [0.1-0.4] | 0.1 [0.1-0.2] | 0.65 | 0.1 [0.0-0.6] | 0.0 [0.0-0.1] | 0.75 | 0.1 [0.0-0.2] | 0.0 [0.0-0.2] | 0.655 | |
| Premature cycling (n/min) | **0.0 [0.0-0.1]** | **0.0 [0.0-0.0]** | 0.32 | **0.0 [0.0-0.0]** | **0.0 [0.0-0.0]** | — | **0.1 [0.1-0.2]** | **0.0 [0.0-0.0]** | 0.25 | |
| Late cycling (n/min) | 0.1 [0.1-0.1] | 0.0 [0.0-0.1] | 0.18 | 0.0 [0.0-0.0] | 0.0 [0.0-0.0] | 0.32 | 0.0 [0.0-0.1] | 0.0 [0.0-0.1] | 0.75 | |
| Data are presented as median [interquartile range]. P: P value calculated between PSV and NAVA using Wilcoxon test. BMI= Body Mass Index; PSV = Pressure Support Ventilation; NAVA = Neurally Adjusted Ventilatory Assist; DI =Dyssynchrony index n/min = Number of events/min; T_d_ inspiratory trigger delay; T_iex_ inspiratory time in excess. | | | | | | | | | |  |

**Table S6**: Variability of ventilatory parameters during each ventilatory period for PSV and NAVA in all patients, with and without obesity.

|  | All patients (n=21) | | | Patients with obesity (n=10) | | | Patients without obesity (n=11) | | |
| --- | --- | --- | --- | --- | --- | --- | --- | --- | --- |
|  | PSV | NAVA | P | PSV | NAVA | P | PSV | NAVA | P |
| RR(%) | **27 [16-38]** | **43 [30-74]** | **0.001** | **31 [22-45]** | **44 [42-49]** | **0.013** | **18 [12-31]** | **37 [28-80]** | **0.037** |
| Ti (%) | **17 [12-24]** | **22 [17-32]** | **0.005** | 18 [12-23] | 27 [18-33] | 0.084 | **16 [9-23]** | **22 [19-29]** | **0.048** |
| Ttot (%) | 21 [16-27] | 24 [19-32] | 0.089 | 22 [18-23] | 25 [20-32] | 0.130 | 18 [11-28] | 24 [19-28] | 0.375 |
| VT (%) | **19 [12-29]** | **24 [20-38]** | **0.007** | **21 [14-21]** | **31 [19-42]** | **0.048** | **15 [10-29]** | **24 [22-28]** | **0.105** |
| MV (%) | **22 [16-32]** | **35 [30-41]** | **0.001** | 27 [18-33] | 39 [34-41] | 0.064 | **18 [12-25]** | **31 [26-40]** | **0.019** |
| Max P_insp_ (%) | **2 [1-2]** | **20 [15-24]** | **<0.001** | **2 [1-2]** | **23 [16-26]** | **0.002** | **2 [1-2]** | **19 [16-23]** | **0.002** |
| Mean P_insp_ (%) | **3 [2-4]** | **15 [10-21]** | **<0.001** | **4 [2-4]** | **16 [11-21]** | **0.003** | **3 [1-3]** | **12 [9-20]** | **0.002** |
| Max EADi (%) | 41 [38-56] | 41 [31-49] | 0.087 | 41 [35-54] | 37 [30-49] | 0.652 | 39 [37-56] | 43 [31-47] | 0.0624 |
| Variability was evaluated by the coefficient of variation. Data are presented as median [interquartile range]. P: P value calculated between PSV and NAVA using Wilcoxon test. PSV = Pressure Support Ventilation; NAVA = Neurally Adjusted Ventilatory Assist; RR = Respiratory Rate; Ti = Inspiratory Time; Ttot = total time of respiratory cycle; VT = Tidal Volume; VM = Minute Ventilation ; Max P_insp_ = Maximal Inspiratory Airway Pressure; Mean P_insp_ = Mean Inspiratory Airway Pressure; Max EADi: Maximal electrical activity of the diaphragm. | | | | | | | | | |

**Figure S1**: Representative tracing of airway pressure, flow, respiratory volume and electrical activity of the diaphragm (EADi) during (A) pressure support ventilation (PSV) and (B) neurally adjusted ventilatory assist (NAVA).


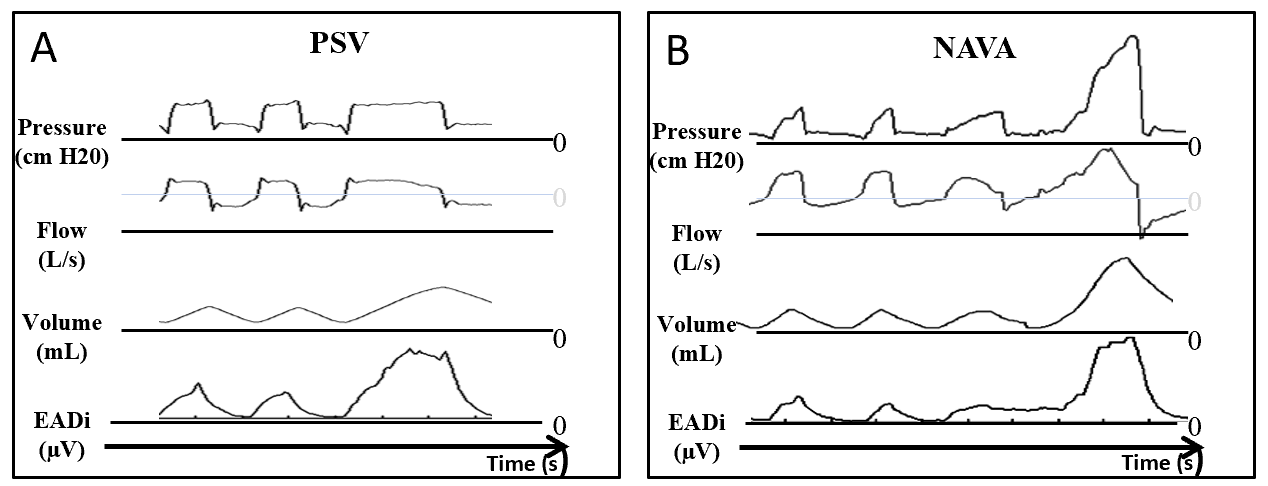


**Figure S2**: Flow chart


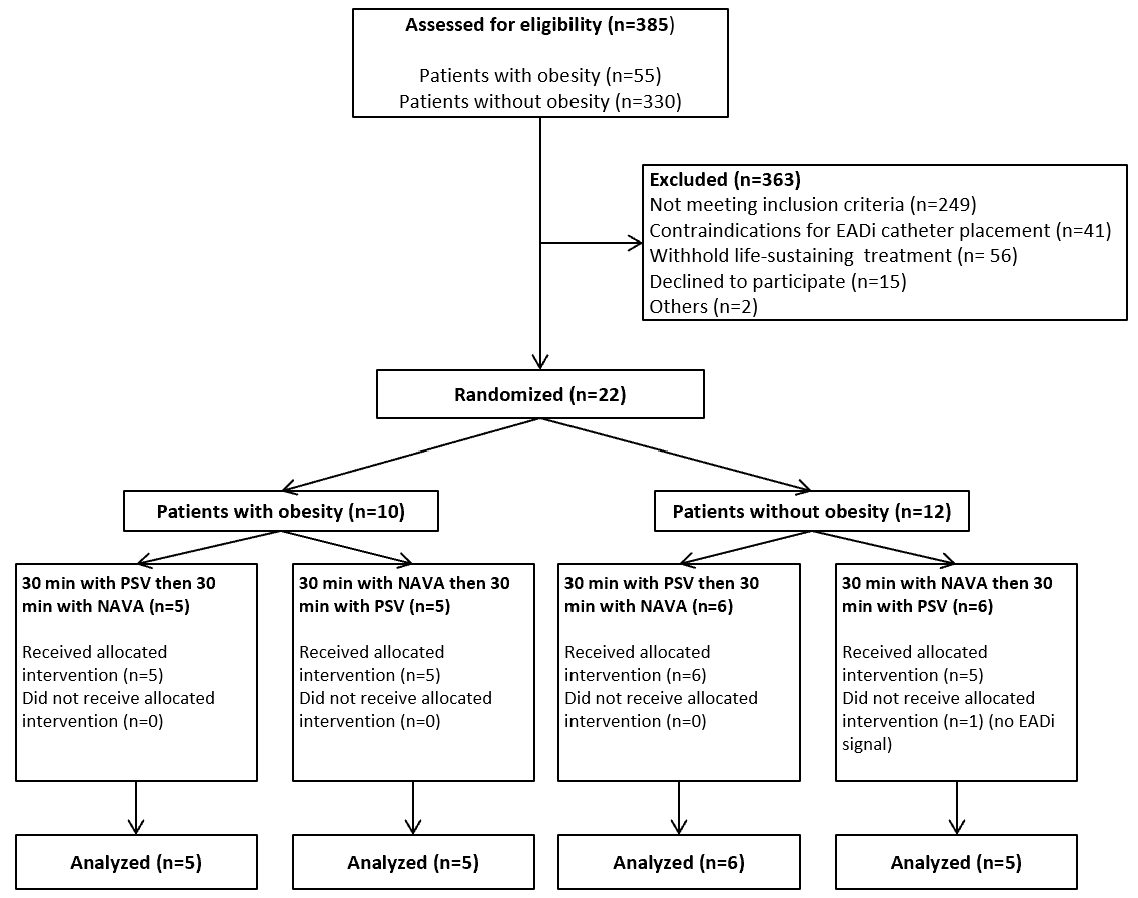


**Figure S3**: Individual percentage of pneumatic cycle with maximal inspiratory airway pressure higher than 30 cmH2O during Pressure Support Ventilation (PSV) and Neurally Adjusted Ventilatory Assist (NAVA) in all patients, with and without obesity. Plots represent individual percentages. The horizontal bars represent the mean values in each group.


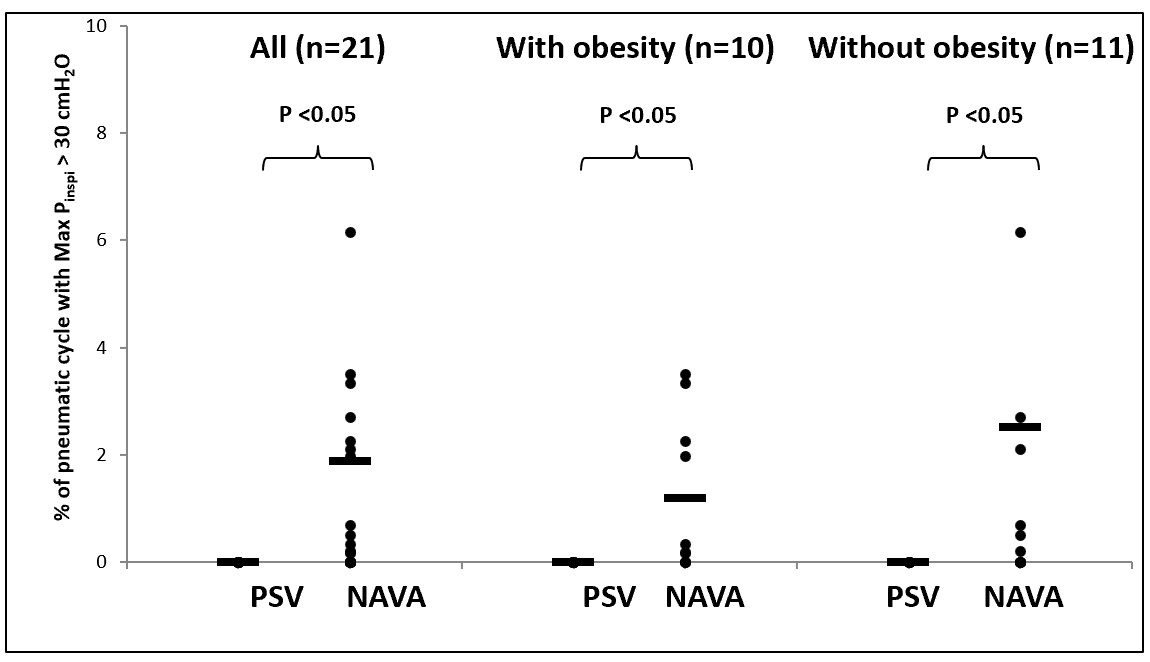


**Figure S4**: Scatter plots of the variation of PaO2/FiO2 ratio according to tidal volume variability (VT variability) (left) or Maximal inspiratory airway pressure variability (Max P_insp_ variability) (right) measured in all patients (top), with obesity (middle) and without obesity (bottom) during pressure support ventilation (PSV) and neurally adjusted ventilatory assist (NAVA).


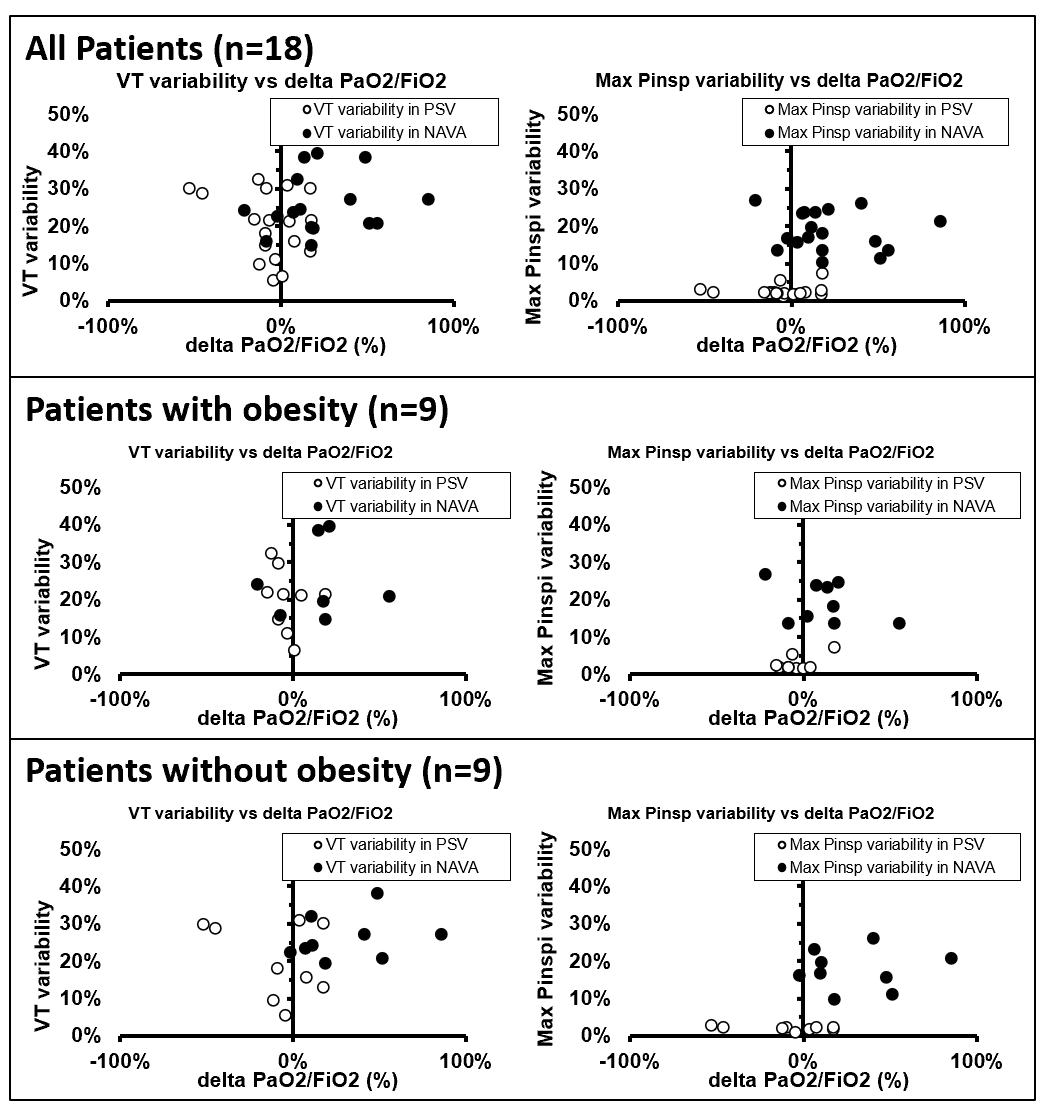


**Figure S5**: Individualized scatter plots of maximal inspiratory airway pressure (Pmax, cmH2O) vs. tidal volume (Vt, mL/kg) measured in patients with obesity during Pressure Support Ventilation. Lines represent linear regression lines.


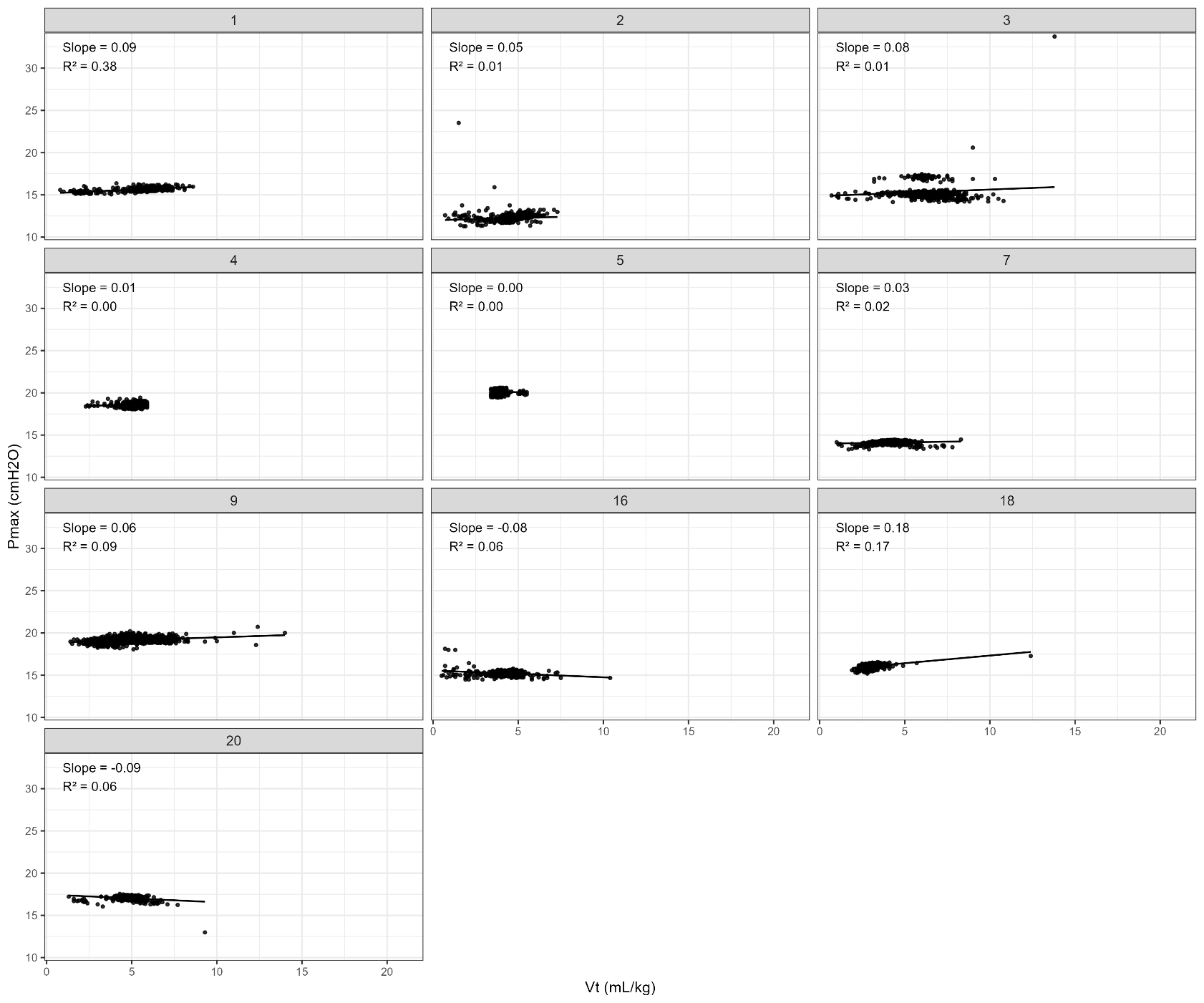


**Figure S6**: Individualized scatter plots of maximal inspiratory airway pressure (Pmax, cmH2O) vs. tidal volume (Vt, mL/kg) measured in patients with obesity during Neurally Adjusted Ventilatory Assist. Lines represent linear regression lines.


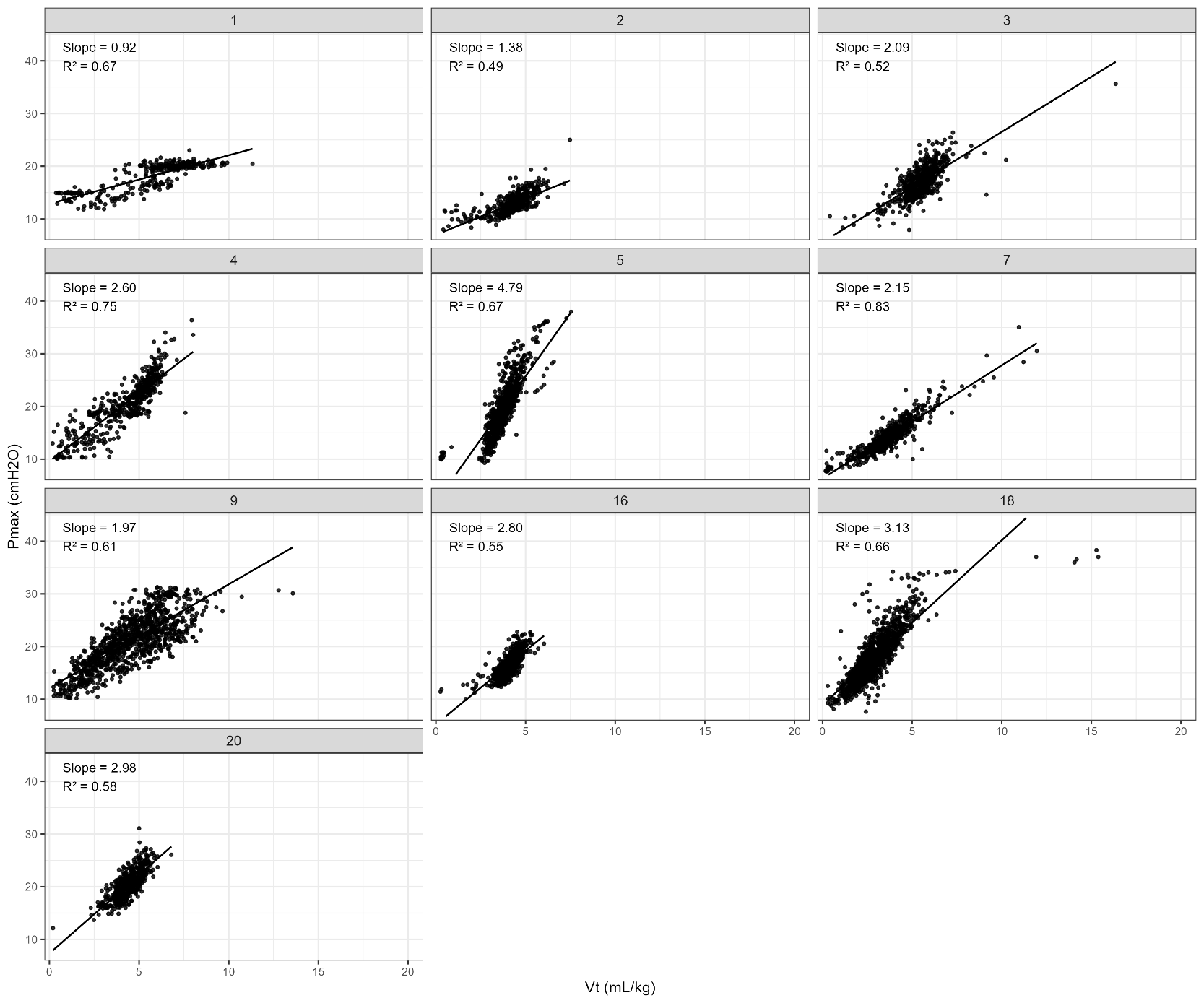


**Figure S7**: Individualized scatter plots of maximal inspiratory airway pressure (Pmax, cmH2O) vs. tidal volume (Vt, mL/kg) measured in patients without obesity during Pressure Support Ventilation. Lines represent linear regression lines.


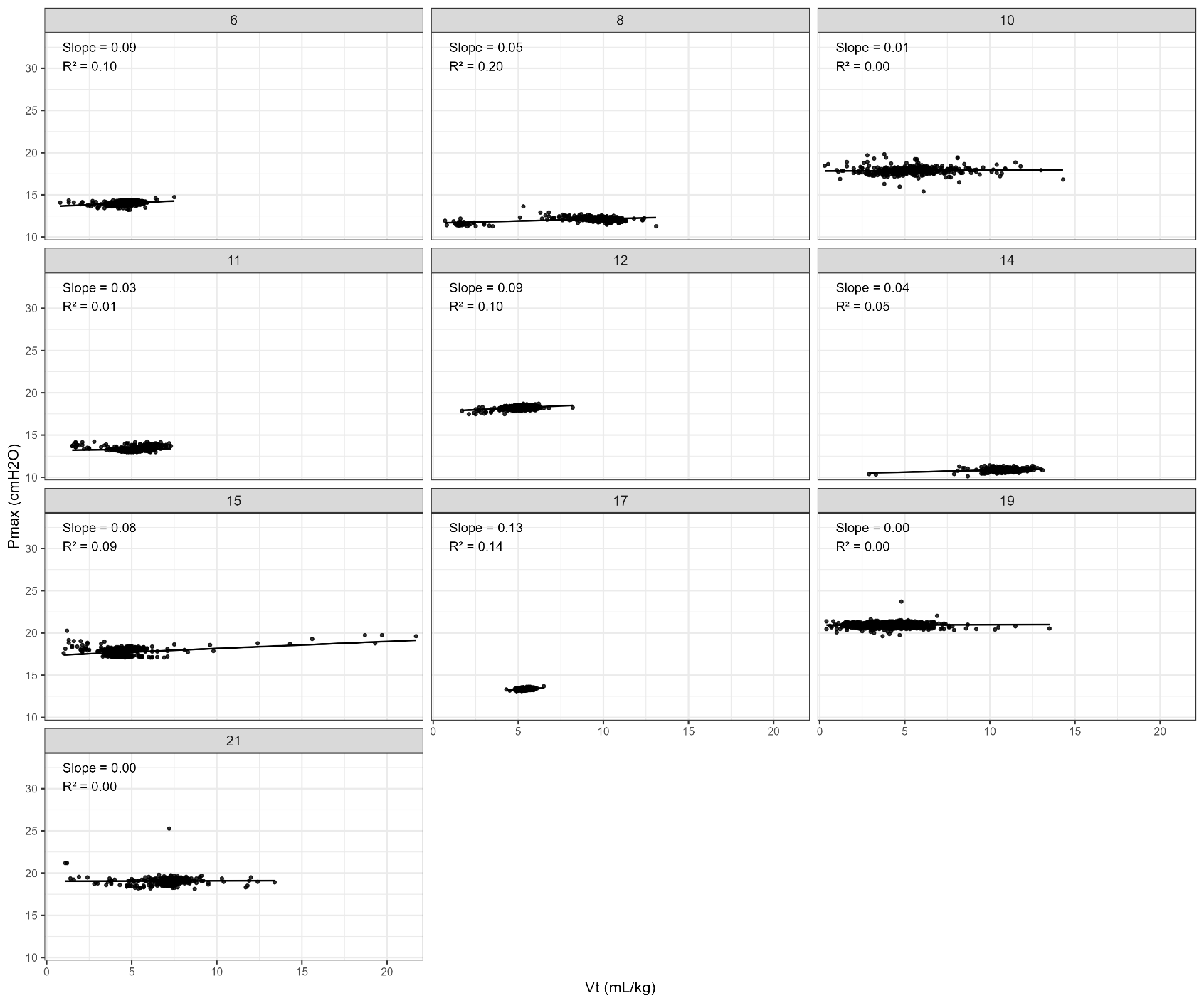


**Figure S8**: Individualized scatter plots of maximal inspiratory airway pressure (Pmax, cmH2O) vs. tidal volume (Vt, mL/kg) measured in patients without obesity during Neurally Adjusted Ventilatory Assist. Lines represent linear regression lines.


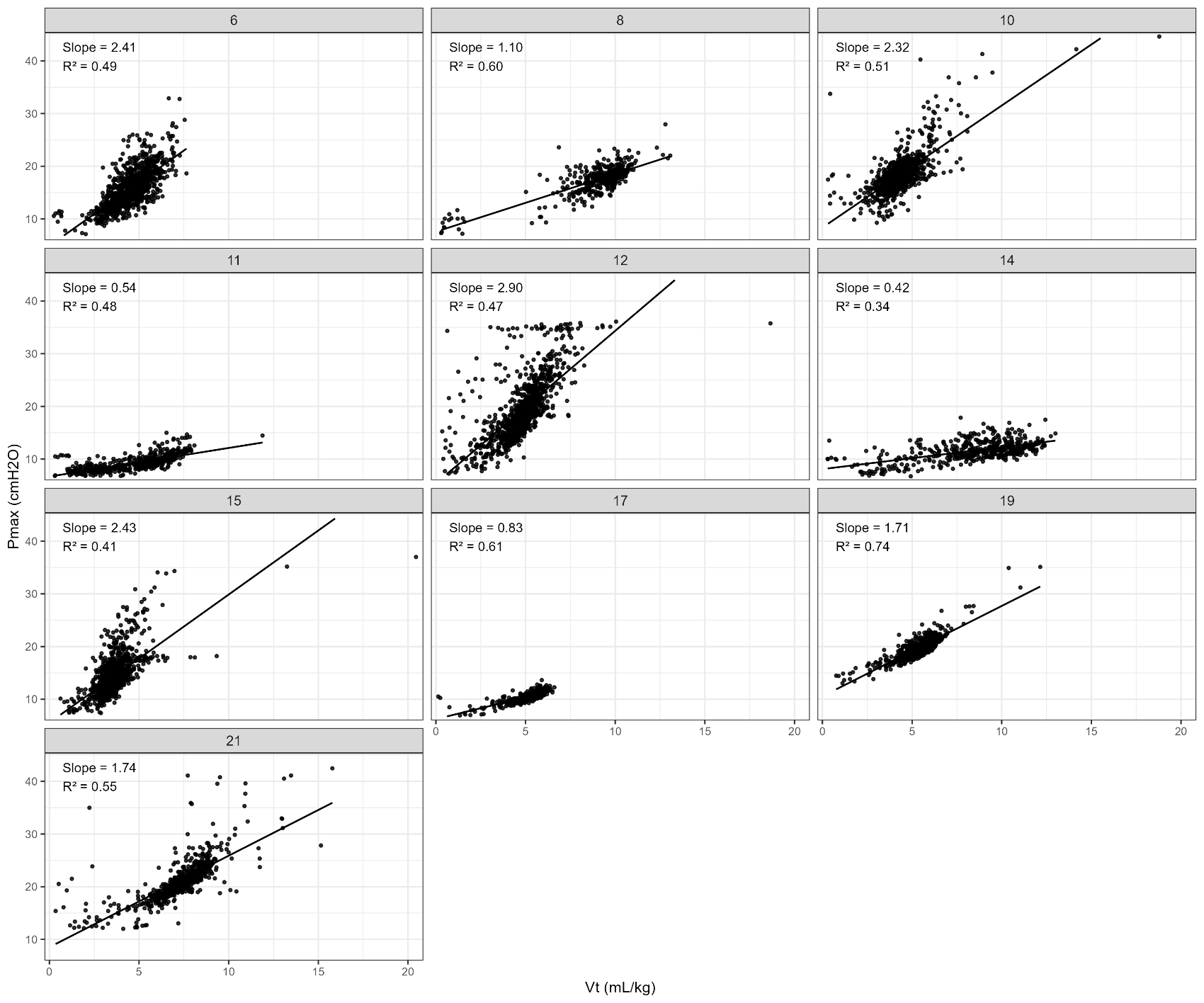

Supplement: Supplementary file 1 — Supplementary Material 1 [file 13613_2025_1552_MOESM1_ESM.docx]
